# Evaluation of head posture using an inertial measurement unit

**DOI:** 10.1038/s41598-021-99459-7

**Published:** 2021-10-07

**Authors:** Mustafa Al-Yassary, Kelly Billiaert, Gregory S. Antonarakis, Stavros Kiliaridis

**Affiliations:** 1 Division of Orthodontics, University Clinics of Dental Medicine, University of Geneva, Geneva, Switzerland; 2 Dept of Orthodontics and Dentofacial Orthopaedics, School of Dental Medicine, University of Bern, Bern, Switzerland

**Keywords:** Dentistry, Medical research

## Abstract

An inertial measurement unit (IMU) is an electronic device that measures and track the orientation of a body. We conducted this study in accordance with the STARD guidelines to evaluate the accuracy of IMU (index test) for measuring head posture compared to the current gold standard using a cervical range of motion (CROM) device. The reproducibility of the hunter and mirror-guided head posture was also evaluated. In vitro and in vivo tests were carried out to assess the validity of the IMU. To assess reproducibility, thirty healthy young adults were asked to look at four different locations in two different sessions while the head posture was recorded. Excellent correlation (r = 0.99; p < 0.001) was found between the IMU and CROM device with an absolute mean difference of 0.45° ± 0.58° (p = 0.85) for the in vitro test and 0.88° ± 1.20° (p = 0.99) for the in vivo test. For the reproducibility test, moderate to good correlation coefficients were found (r = 0.55 to 0.89; all p < 0.05) between the two sessions. The intraclass correlation coefficient ranged from moderate to excellent reliability (ICC from 0.74 to 0.96). These results suggest that the IMU sensors, when calibrated correctly, can be adequate to analyze head posture.

## Introduction

Natural head posture (NHP) is defined as “when a man is standing and when his visual axis is horizontal, his head is in a natural position”, first introduced by Broca^1^. Studies have shown that the head posture has been correlated to craniofacial morphology^2,3^, malocclusion^4^, facial growth pattern^5^, respiratory^6,7^, and ophthalmology^8,9^ alterations. However, those studies use different methods to measure the head postures making it difficult to compare the results between studies. It would be interesting to find a simple, fast, inexpensive and precise technique to measure the posture of the head in the 3 axes.

Different methods exist to analyze head posture. The subjective method is an interpretation by the operator to assess the NHP^10^. For research purposes, where an objective assessment of head posture is sought, there are three types of methods to assess head posture:

*Head-mounted equipment* This is the standard method to assess head posture, which can be considered as the gold standard. The Cervical Range of Motion (CROM) device is a good example^11^, but there can be faulty readings if the device has been placed incorrectly or if the patient has facial asymmetry. Moreover, when installing the device, the patient may adopt a different position due to the weight of the device. Head-mounted equipment can also be used with electronic gyroscopes.

*Two-dimensional photographic analyses* The use of photographs is another method to assess the NHP. It is simple, fast, inexpensive, and gives accurate results. Unlike the previous method, it does not require the patient to wear any bulky equipment. However, the axis of the photograph must be very precise, and it can be difficult to adopt with patients that are moving^12^. The same principle can be used in radiology, but it exposes the patient to unnecessary radiation.

*Three-dimensional (3D) analyses* 3D optic scans of the head can also be used to measure the NHP. The patient does not have to wear any equipment and some systems have very high precision. The patient however has to stay still for a certain period without any movement, and this may introduce some bias. This method needs a complex setup, is expensive, and requires a good knowledge of the associated software^13^.

To perform reproducible analyses, there are three standards postures, the self-balanced, the self-guided, and the hunter position. The self-balanced head posture is adopted when a person looks at an imaginary point in front of them, from an infinite distance at eye level^13^. The self-guided head posture is when a person looks at themselves in a mirror^2^. The hunter position is adopted when a person looks at a certain point^8^. The head posture can be evaluated along three axes: pitch, roll, and yaw.

In recent years, wearable sensors composed of an accelerometers and gyroscopes, commonly called inertial measurement units (IMUs), have become increasingly popular to measure human motion. IMU allows to measure joint angles using the orientation in space collected from two sensors^14,15^. Magnetic angular rate and gyroscope sensors can also be part of the IMU, the additional reading of the earth’s magnetic field allows to have more accurate orientation in 3D^16^. The benefits of this system are its ease of use, simplicity, and affordability. Our goal was first to analyze the validity of the IMU system in vitro and then in vivo for measuring the head posture. We also wanted to estimate the variation of head posture in a group of healthy young adults.

## Methods

### Ethics statement

The present study was approved by the Swiss Association of Research Ethics Committees. The experimental procedures were conducted in conformity with the Declaration of Helsinki. Informed consent for participation in the study and publication in an open access format was obtained from the participants, with regard to their photographs and personal information. The procedures of the study were fully explained to the participants and they provided their informed written consent before testing.

### Material

For the present study, the MetaMotionR (MbientlabInc., San Francisco, CA, USA) was used as the IMU. This system is composed of two sensors. This system uses cloud storage to allow health professionals to monitor the patients. Calibration is required prior to use, and this is carried out by positioning the sensor in a neutral position (corresponding to 0° on each axis).

**Figure 1:**
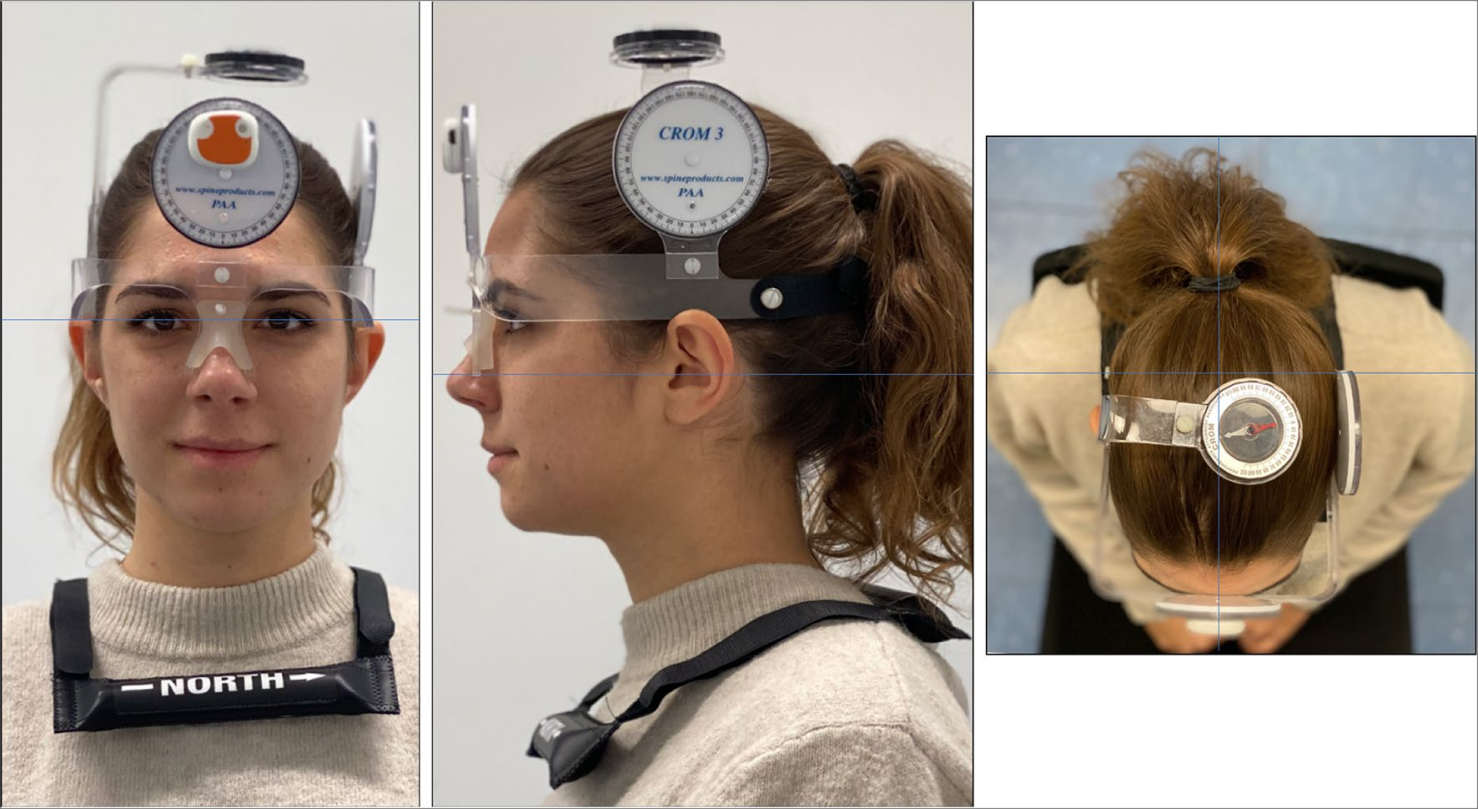
Photograph of the setup used for the in vivo assessment of validity of the inertial measurement unit. The cervical range of motion device was placed on the head with the first sensor fixed on the roll inclinometer and the second one placed on the ground acting as the reference.

We followed the recommendation of the STARD guidelines^17^ for reporting the diagnostic accuracy of the index test (IMU) compared against the reference standard (CROM device). Investigations of validity were performed both using in vitro and in vivo models. The CROM device is considered the best available method to date to assess head posture (gold standard). This device is placed on the head like glasses, and includes two inclinometers, one located on the forehead and the other on the temporal bone. This allows for the assessment of pitch and roll. A compass is located at the top of the head and along with the magnets on the shoulders this allows for the assessment of the yaw (Fig. 1).

### In vitro assessment of validity

**Figure 2:**
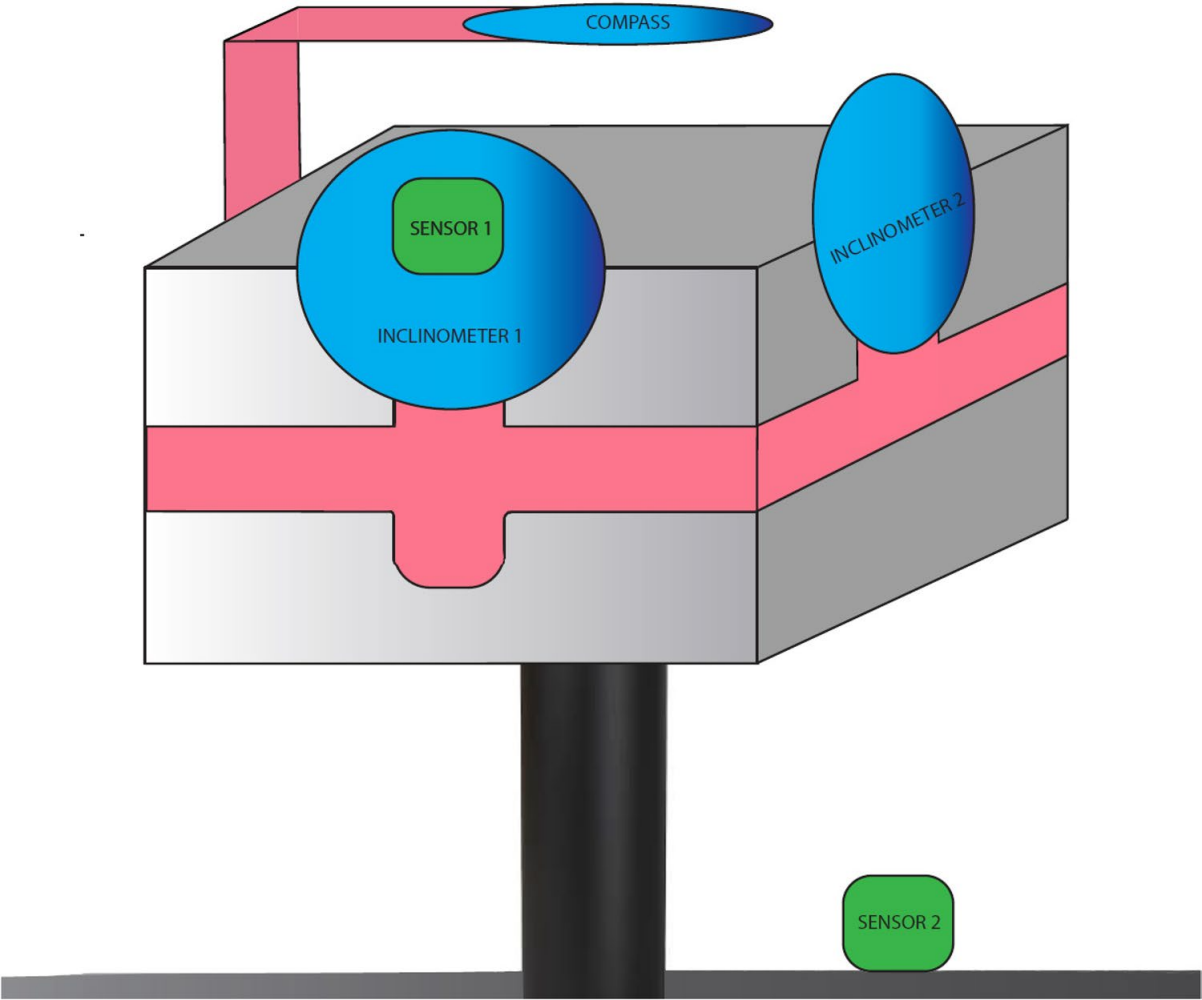
Representative image for the in vitro assessment of the inertial measurement unit. A box-shaped model (grey) was used with the cervical range of motion device (pink and blue) placed on top. The first sensor (green) was placed on the inclinometer 1, and the second on the table. It was possible to move and block this model on the 3 axes separately.

The validity of the method was first investigated using an in vitro model. A box-shape model was used as the representation of the head, which could be moved and blocked on the three axes separately. Initial calibration of the IMU was carried out when the readings from the CROM device on the three axes were 0°. Two IMU sensors were used, one placed on the front inclinometer and the second placed on a table behind it (Fig. 2). The model was subsequently moved, one axis at a time, guided by the CROM device. The degrees were increased by 10° sequentially until 90° were reached. The same methodology was used for the negative degrees (from 0° to − 90°). At each 10° interval, measurements were recorded using the IMU. The in vitro assessment was carried out on two separate occasions, with a one-week interval between sessions. In total 60 recordings (20 for each of the three axes) were taken with the CROM device and the IMU at each session.

### In vivo assessment of validity

The validity of the method was investigated with five healthy collaborators at the University clinics of dental medicine in Geneva, Switzerland. The human subjects wore the CROM device on their head and the IMU sensor was placed on the front inclinometer. The second sensor was placed on the floor to act as the reference, using the same concept as in the in vitro model. The IMU was calibrated when the readings from the CROM device on the three axes were 0°. The subjects were subsequently asked to move their head on each axis separately. The analysis was carried out with a sequential increase in each axis by 10° up to the maximum. At each interval, the reading from the IMU was recorded (Fig. 1). In total, 217 recordings were transcribed (ranging from 48 to 39 recordings per person).

### Variation of head posture and reproducibility assessment

**Figure 3:**
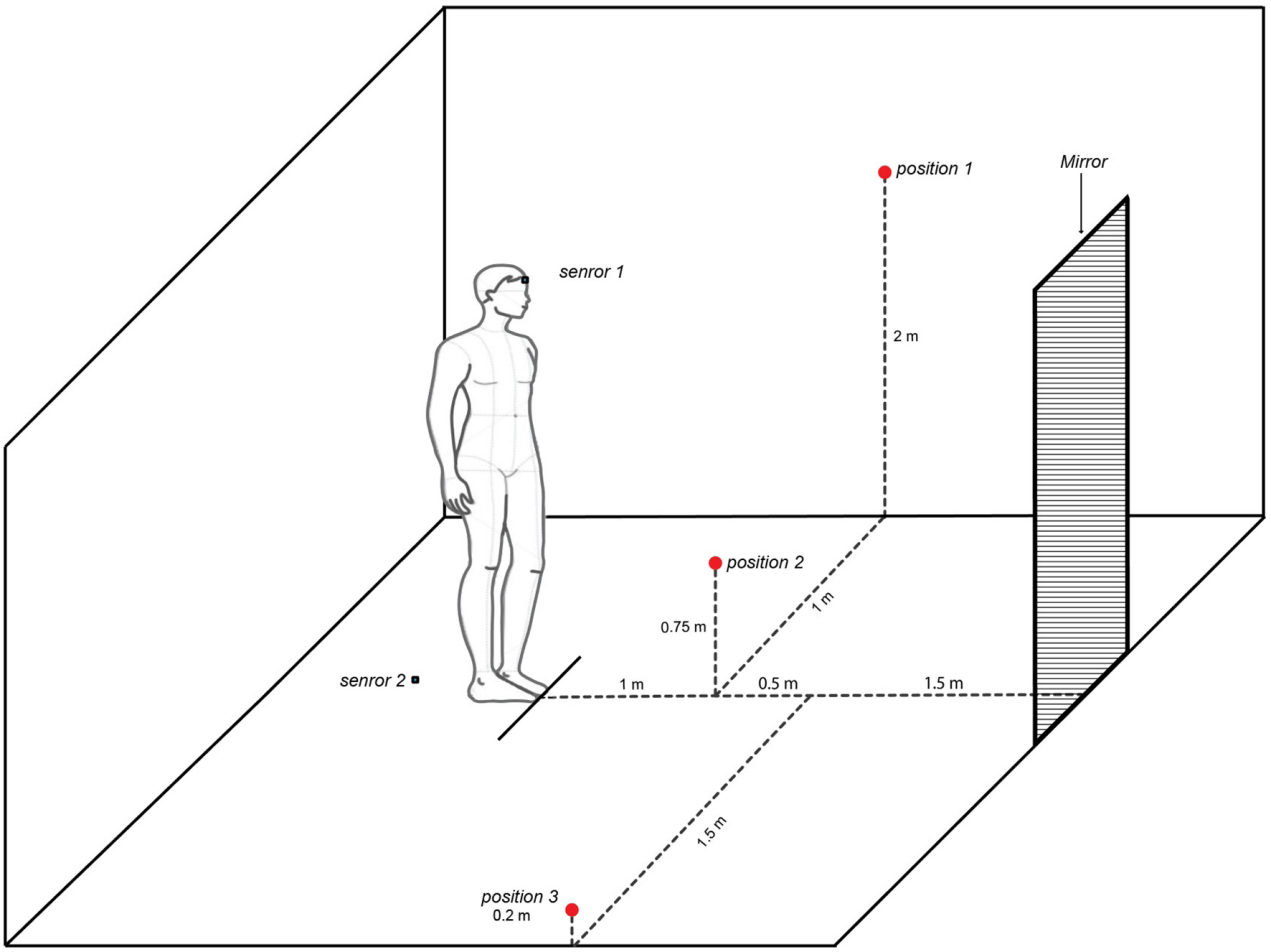
Representative image for the assessment of head posture and the reproducibility of the inertial measurement unit system. The participants were positioned in front of a mirror located 3 m in front of them. The first point was located 90 cm in front, 1 m to the left, and 2 m above their feet (position 1). The second point was 90 cm in front and 75 cm in height (position 2). The third point was located 1.50 m in front, 1.50 m to the right, and 20 cm in height (position 3).

A group of 30 healthy young adults, all collaborators of the University clinics of dental medicine in Geneva, Switzerland, were used to assess the variation of head posture and the reproducibility of the method. They were asked to look at four different locations, while standing (orthoposition^18^) in a well-illuminated room. This included a mirror located three meters in front of them where they were asked to look at themselves, as well as three different points which were indicated with a number and a point (0.5 cm of diameter) (Fig. 3). Head posture was recorded with the IMU as the participant looked at each of these four locations. Calibration of the IMU was carried out with the participants looking at their eyes in the mirror (mirror guided head posture), with one sensor on the forehead and the other on the floor used as the reference. Following this calibration, the participants were asked to look at each of the three points in order, looking at the mirror in-between each of the three positions. This was done twice for each position during the same session. The same procedure was carried out on two separate occasions, with a one-week interval. In total 20 recordings were carried out for each participant (10 in each session, namely two for each position and four for the mirror).

### Statistical analyses

All statistical analyses were performed using SPSS (version 24.0, SPSS Inc., Chicago, IL, USA). To compare the results of the IMU system with the CROM device, paired t-tests were used as well as Pearson correlation coefficients for each position. Linear regression analysis was performed for the in vitro and in vivo test with the results from the CROM device as the independent variable, and those from the IMU as dependent variable. Intraclass correlation^19^ estimates and their 95% confident intervals (CI) were calculated^20^. ICCs performed for the in vivo and in vitro tests were based on a single rating, absolute-agreement, 2-way random-effects model, while for the variation in head posture and reproducibility ICCs were performed based on a mean-rating (k = 4), absolute-agreement, 2-way mixed-effects model. ICC values less than 0.5 are indicative of poor reliability, values between 0.5 and 0.75 indicate moderate reliability, values between 0.75 and 0.9 indicate good reliability, and values greater than 0.9 indicate excellent reliability^21^. The standard error measurement (SEM) of the difference between session 1 and 2 was calculated for each ICC (using the formula SEM = $\mathrm{Standard Deviation }\times \sqrt{1-\mathrm{ICC}}$)^20^.

For the in vivo test, a Bland–Altman plot was made to visualize the spread of the differences between the CROM device and the IMU for each recording compared to their mean. Bland–Altman plots were also used to visualize the spread of the differences between the two sessions for each individual axis.

## Results

### In vitro assessment of validity

The mean of the absolute difference between the CROM device and the IMU in vitro was 0.45° ± 0.58° (p = 0.85). A strong correlation was observed between the two systems (r = 0.99, p < 0.001). For every axis and between the two sessions, no systematic differences between the two systems were reported (p > 0.05). The ICC between the CROM device and IMU was excellent (ICC = 1.000) with a 95% CI between 0.999 and 1.000 and a SEM of 0°.

### In vivo assessment of validity

The mean of the absolute difference between the CROM device and the IMU in vivo was 0.88° ± 1.20° (p = 0.99). A strong correlation was observed between them (r = 0.99, p < 0.001). For each axis no systematic differences between the two systems were reported (p > 0.05). Unlike for the in vitro study the yaw was slightly less precise than the pitch and the roll (Table 1). Even with extreme head postures, an increase in the variation between the CROM device and the IMU was not observed. The ICC between the CROM device and the IMU was excellent (ICC = 1.000) with a 95% IC between 0.999 and 1.000 and a SEM = 0°.

**Table 1:** Summary table comparing the inertial measurement unit to the cervical range of motion device.

	Total	Pitch	Roll	Yaw
**In vitro**				
Mean ± SD	0.45 ± 0.58	0.47 ± 0.62	0.38 ± 0.50	0.50 ± 0.49
r (p value)	0.99 (0 < 0.001)	0.99 (< 0.001)	0.99 (< 0.001)	0.99 (< 0.001)
**In vivo**				
Mean ± SD	0.88 ± 1.20	0.61 ± 0.80	0.80 ± 1.04	1.28 ± 1.61
r (p value)	0.99 (< 0.001)	0.99 (< 0.001)	0.99 (< 0.001)	0.99 (< 0.001)

The mean and standard deviation (SD) of the differences represents the average of the absolute difference between the cervical range of motion device (gold standard) and the inertial measurement unit. The Pearson correlation (r) with the respective p value is also shown.

**Figure 4:**
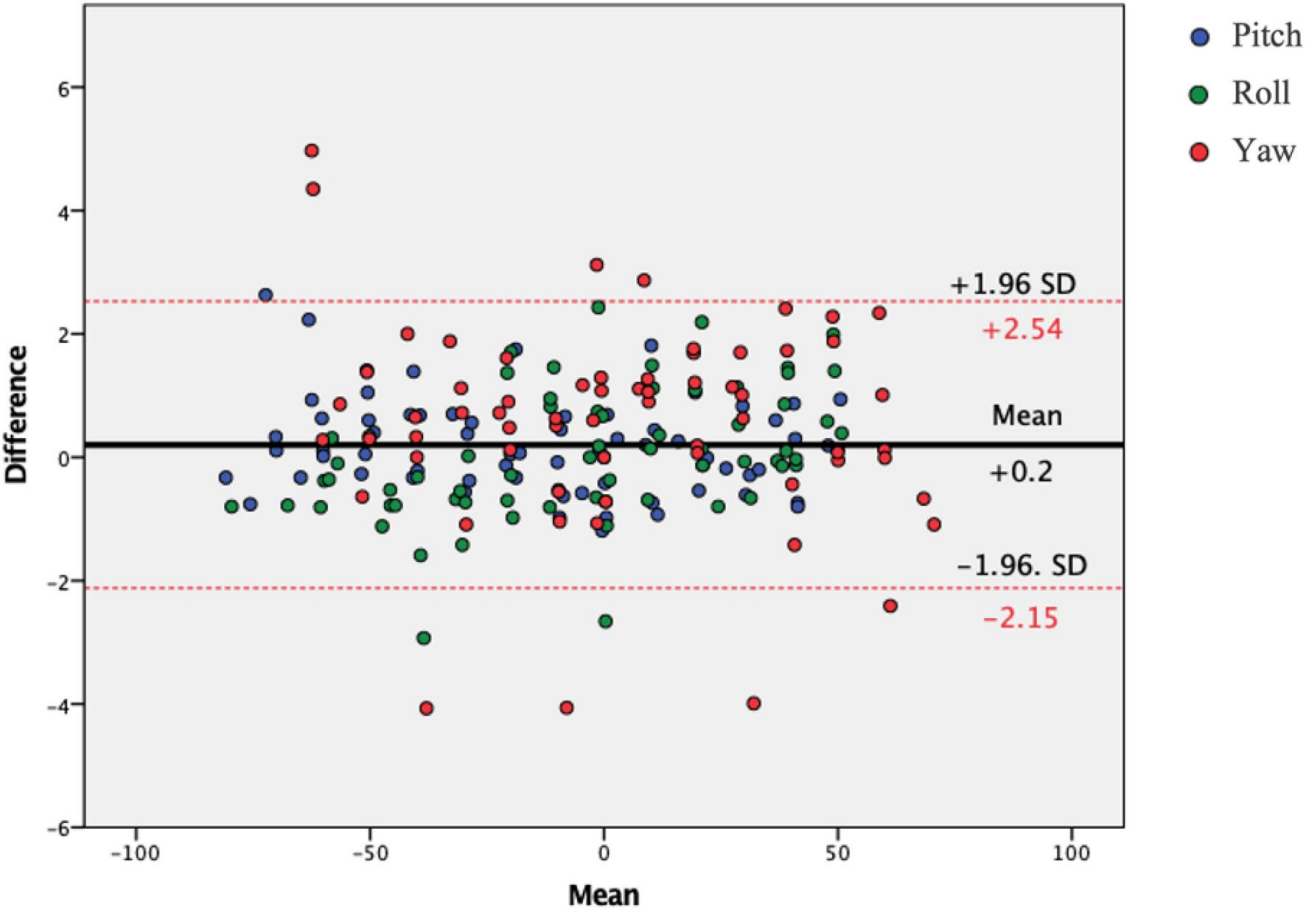
Bland–Altman plot between the cervical range of motion device and inertial measurement unit in vivo The X axis represent the mean between the cervical range of motion device and the inertial measurement unit and the Y axis the mean differences between them. Red lines indicate the 95% confidence intervals, and the black line indicates the mean of the differences. Blue points represent the Pitch, green points the Roll and red points the Yaw.

The Bland–Altman plot shows the mean differences at + 0.2° and the 95% CI between − 2.15 and + 2.54. No systematic bias is observed, with the data points being distributed equally below and above the mean line. Most of the points situated outside the 95% CI are Yaw measurements (Fig. 4).

### Variation of head posture and reproducibility

The most stable axis (with the smallest SD) was found to be the Roll, followed by Yaw and finally Pitch (Table 2). For every axis and between the two sessions, no systematic error was found (p > 0.05). The ICC between all eight recordings for the mirror position had moderate to good reliability (ICC between 0.74 and 0.96) depending on the axis (Table 2).

**Table 2:** Characteristics of the natural head position achieved in front of a mirror and each of three hunter positions.

	Mean ± SD	ICC	95% CI	SEM
**Mirror**				
Pitch	− 0.31 ± 2.34	0.74	0.57–0.86	1.20
Roll	0.35 ± 1.22	0.82	0.70–0.90	0.52
Yaw	0.41 ± 1.83	0.84	0.74–0.91	0.73
**Position 1**				
Pitch	− 7.00 ± 3.98	0.89	0.81–0.94	1.32
Roll	− 2.65 ± 4.30	0.92	0.86–0.96	1.24
Yaw	32.88 ± 5.35	0.73	0.53–0.86	2.77
**Position 2**				
Pitch	26.48 ± 7.54	0.95	0.91–0.97	1.75
Roll	1.62 ± 2.36	0.85	0.73–0.92	0.92
Yaw	− 3.57 ± 2.45	0.83	0.71–0.91	1.00
**Position 3**				
Pitch	15.17 ± 5.67	0.96	0.92–0.98	1.19
Roll	− 7.71 ± 4.44	0.93	0.89–0.97	1.15
Yaw	− 36.41 ± 5.72	0.94	0.90–0.97	1.39

Means and standard deviations (SD) are shown for each position and each axis within each position, for the 30 participants. Intraclass correlation^19^, 95% confidence intervals (CI), and standard error measurements (SEM) are also shown.

**Figure 5:**
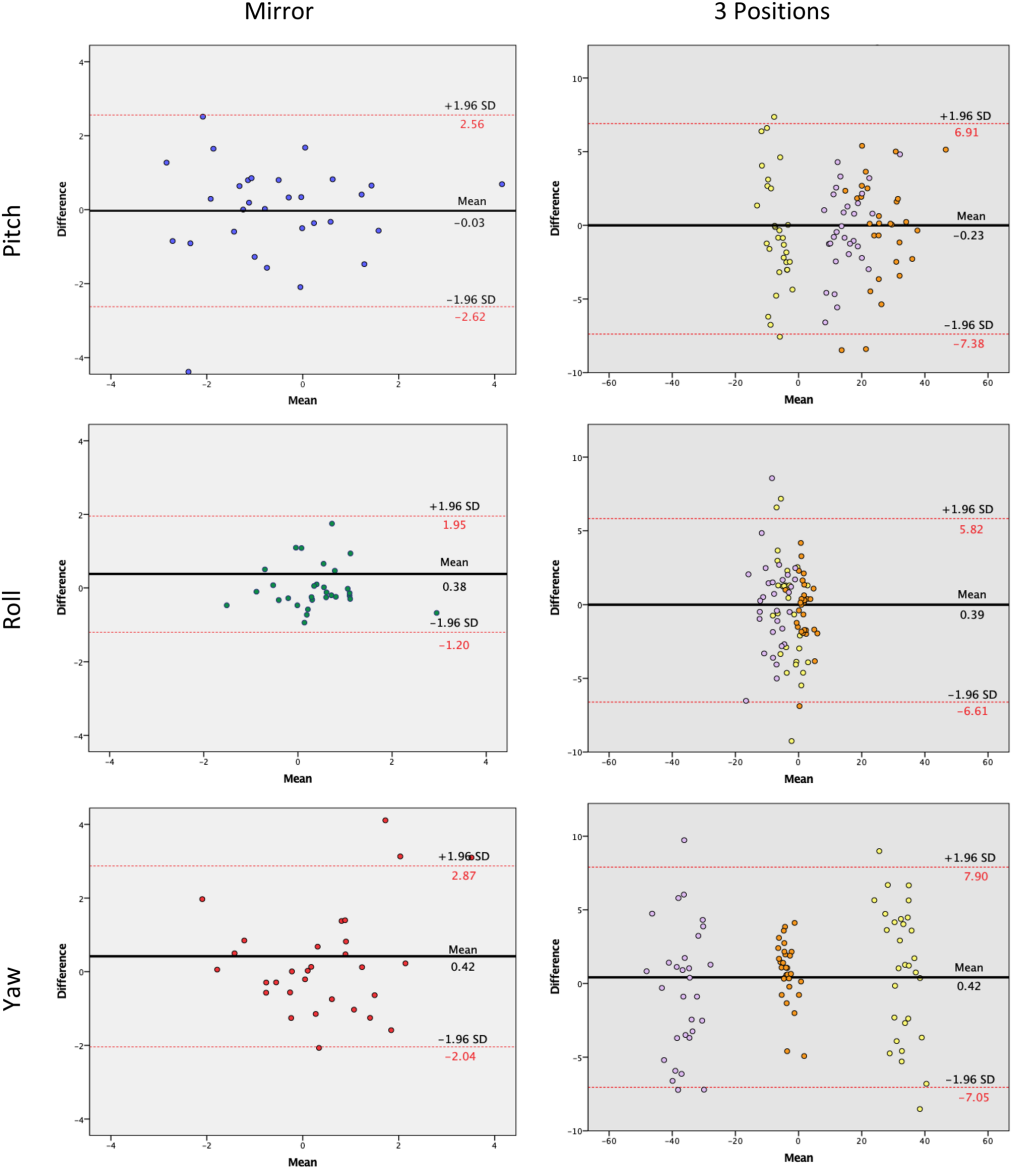
Bland–Altman plot between the two session: for mirror and the three positions. Bland–Altman plots show the mean differences in the measurement of the natural head position using the mirror (left column) and the 3 hunter positions (right column) from the 30 participants. Red lines indicate the 95% confidence intervals while black line indicate mean of the differences. For the 3 positions, yellow points indicate the first position, orange points the second position and purple points the third position.

The Bland–Altman plot shows the distribution of the difference between the two sessions. The mean of the first four recordings for the mirror position were calculated and compared to the mean of the last four recordings. The X axis represent the mean between the two sessions and the Y axis the differences between them (Fig. 5). The mirror position shows a higher stability than the hunter position with 95% CI < 3° for the mirror and 95% CI > 5.5° for the hunter positions. Overall, the distribution is equal above and below the mean.

For the hunter position, four recordings were measured for each position (two during each session). A greater SD was observed compared to the mirror position but with better reliability (from moderate to excellent) (Table 2). Overall, there was no systematic difference between the first and second session (p > 0.05).

To better understand the stability and reproducibility specific to each participant the measurements from the two sessions were compared. For the three hunter positions, the difference between the mean of the first two recording (done the same day) and the last two recordings (done after one week) was calculated. Significant intra-individual variation was observed. The roll seems to be the most precise and stable axis, followed by pitch, and finally yaw with the largest amount of variation (Table 3). Paired T-test showed no systematic difference between the first and second sessions (p > 0.05).

**Table 3:** Difference between the two sessions Differences, presented as absolute mean and standard deviation (SD), between the mean of the 1st and 2nd recording (done in the first session) and the mean of the 3rd and 4th recording (done in the second session) for each axis are shown.

Position		Mean ± SD	r (p value)
1	Pitch	2.41 ± 3.31	0.64 (< 0.001)
	Roll	2.60 ± 3.40	0.70 (< 0.001)
	Yaw	3.90 ± 4.49	0.57 (< 0.001)
2	Pitch	2.97 ± 4.19	0.84 (< 0.001)
	Roll	1.56 ± 2.14	0.55 (0.002)
	Yaw	1.78 ± 2.11	0.61 (< 0.001)
3	Pitch	2.27 ± 2.83	0.89 (< 0.001)
	Roll	2.33 ± 3.05	0.75 (< 0.001)
	Yaw	3.53 ± 4.35	0.66 (< 0.001)

Pearson correlation coefficients (r) and p value are also shown.

## Discussion

### Assessment of validity

The present study has shown that there is an excellent correlation in vitro as well as in vivo between the CROM device and the IMU. Our results on head posture are similar to the ones found previously by Beange et al.^22^ who compare the MetaMotionR to a 7-camera motion capture (Vicon MX40) on the spine with an excellent degree of similarity. The yaw is slightly less precise than the two other axes. It is mainly due to the fact that the pitch and the roll are given by two inclinometers but the yaw is given by compass on the CROM device. The compass has a larger scale (every 2° for the compass and every 1° on the inclinometers).

The difference between the two systems is slightly bigger in the in vivo test. Two important potential reasons are that the patient cannot remain completely stable, and that the data readings given by the CROM device are more complicated to report. These reasons are operator and patient dependent and are not directly related to the sensors themselves. After using both systems, the authors feel that the IMUs are easier to use because they allow to measure the head posture on the 3 axes simultaneously and do not rely on the human readings of the data and thus eliminate bias in this regard. Furthermore, the sensors are very light weight and not bulky, unlike the CROM device, and this allows measurements of the natural position of the patient to not be influenced by the equipment worn. However, unlike the CROM that can be used directly on the patient’s head, the IMU system needs to be calibrated before the measurement in order to give accurate results.

### Assessment of reproducibility

When looking at a point (hunter's position), three main parameters may influence the posture of the head, namely the posture of the body^23^, the gaze of the eyes^24,25^, and location in the field of vision^26^. When asking the participant to maintain a straight body posture, without moving their eyes and keeping the point in the center of the field of view, it is likely that small head and eye movements can still take place which can explain variations between the two sessions. This variation between sessions is however interesting to study. It was observed that participants with a poor correlation in the first session also had a poor correlation in the second session and vice versa. This can lead to hypothesize that the stability of the posture of the head is very specific to each individual. In addition, it was noticed that in a group of healthy patients, the variation is greater with the rotation and the pitch, but the roll is very stable. Regarding the mirror-guided posture, our results are very similar to previous studies regarding the pitch. Solow and Tallgren^2^ found that the NHP which refers to the mirror-guided head posture is very reproducible with a range of 1.4°. That is very close to our findings (with a range of 2.3°). We also noticed that there are variations detected for the roll and yaw axes.

If we take into consideration the different factor that can influence head posture during the hunter position, we can affirm that this IMU system has a good reproducibility compatible to carry out advanced research in the field.

### Limitations

These results must be interpreted with caution because the population of our study concerns only healthy young adults, with an analysis of NHP in a standing position. It would be interesting to evaluate the posture of the head in a larger population and with different position (such as the sitting position for example).

## Conclusion

IMU sensors have an excellent correlation with the physical head-mounted equipment (CROM device) both for in vitro and in vivo tests. When calibrated correctly, IMU sensors can be a reliable method to analyze head posture. Variations in NHP are approximatively 2° for the pitch and slightly less for the roll and the yaw axes. Stability of head posture is specific to each person and varies depending on the axis.

## Data Availability

All data included in this study are available upon reasonable request from the corresponding author.
